# Wilson’s disease presenting with unusual radiological features

**Published:** 2015-07-06

**Authors:** Shivraj Goyal, Surekha Dabla, Bhuwan Sharma, Jasminder Singh, Kapinder Yadav

**Affiliations:** ^1^Department of Medicine, PT B.D. Sharma Postgraduate Institute of Medical Sciences, Rohtak- 124 001, Haryana, India

**Keywords:** Kayser–Fleischer Ring, Wilson Disease, Brain MRI, Ceruloplasmin, Copper

Wilson’s disease (WD) is an inherited disorder of copper metabolism. It results in copper deposition in toxic concentrations in liver, brain, eye, etc. Radiological features in the form of extensive gray and white matter abnormalities are rare. Here we report a case of WD presenting with encephalopathy and unusual radiological features.

A 26-year-old male, born out of non-consanguineous marriage, presented with insidious onset difficulty in walking and sitting since 6 months and difficulty in speaking since 2 months. Patient was all right 6 months back, when gradual decline in academic performance and inability to carry out day-to-day activities with marked slowness was noticed. His past history was uneventful. There was no family history of similar complaints.

On general physical examination, he had stuporous look and vacant stare. He comprehended vocal commands but was unable to vocalize. Motor examination showed generalized dystonia, exaggerated deep tendon reflexes, and a positive bilateral Babinski’s sign. The Kayser–Fleischer ring was visible on both sides by the naked eye, which was confirmed on slit lamp (Figure 1). On Abdominal examination, there was no hepatosplenomegaly. Chest and cardiac examination was normal.

On laboratory examination, complete blood count, total serum bilirubin, total serum protein, serum transaminases, and alkaline phosphatase showed no abnormalities. Viral serologies for human immunodeficiency virus, hepatitis B antigen, anti-hepatitis C virus Ab were negative. Upper gastrointestinal endoscopy was also within normal limits. His serum ceruloplasmin was decreased to 0.10 g/l (normal 0.20-0.60 g/l), serum copper level slightly raised to 141.25 g/dl (normal 70-140 g/dl), and his 24 h urine copper excretion was increased to 541.68 μg (normal 24 h urine excretion 20-50 µg).

**Figure 1:**
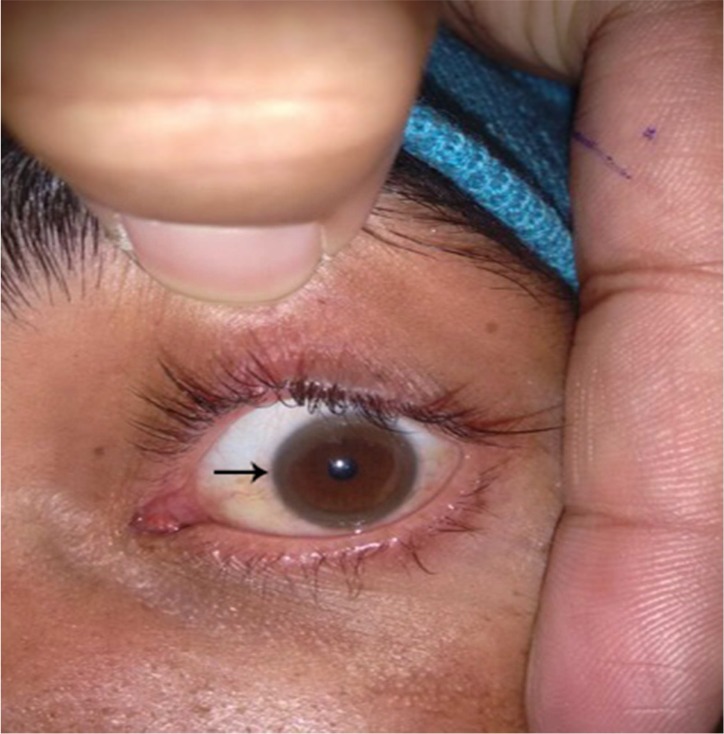
Kayser–Fleischer ring

His ultrasonography (USG) abdomen showed liver with coarse altered echo texture, portal vein diameter 10 mm at formation. Non-contrast computed tomography head showed hypodensity over bilateral white matter region. Findings on magnetic resonance imaging (MRI) brain revealed symmetrical hyperintensity on T2-weighted and fluid attenuated inversion recovery images over bilateral thalami, basal ganglia, claustrum, and dorsal mesencephalon with hypointensity at red nucleus. These hyperintense regions were hypointense on T1-weighted and diffusion weighted images. There was white matter T2 hyperintensity in the bilateral frontal white matter. There was gyriform enhancement in the bilateral frontal region. “Giant Panda face” sign was also present (Figure 2).

WD is an autosomal recessive disorder of copper metabolism. It is caused by a mutation in the copper transporting gene, ATP7B.^1^

An absent or a reduced function of the ATP7B protein leads to a decreased hepatocellular excretion of copper into bile. Copper first accumulates in the liver; after the liver storage capacity for copper gets saturated, copper gets redistributed, with accumulation in the nervous system, cornea, kidney, and other organs.^2^ In WD with neurological presentations, the symptomatology is predominantly extrapyramidal, like dystonia, tremors, dysphasia, dysarthria, and ataxia. The neurological symptoms are secondary to cerebral copper deposition.

In the presence of typical neurological features, ophthalmological features, low serum ceruloplasmin, and increased 24 urinary copper levels. Liver biopsy is not required for the diagnosis of WD (Table 1).

In WD patients, abnormalities are noted in the gray matter of lentiform, caudate, and thalamic nuclei. Cerebral atrophy of frontal lobes and cerebellar atrophy have been described.^3^ Our patient had gray matter abnormalities in bilateral thalami, pons, and red nucleus.

The gray matter abnormalities are manifested as hypodensities in computerized tomography head and as hypointensities on T1 and hyperintensities on T2 images of MRI brain. The findings on MRI brain can be due to underlying gliosis and necrosis.^3^

Our patient had extensive white matter abnormalities in bilateral frontal and parietal regions.

**Figure 2:**
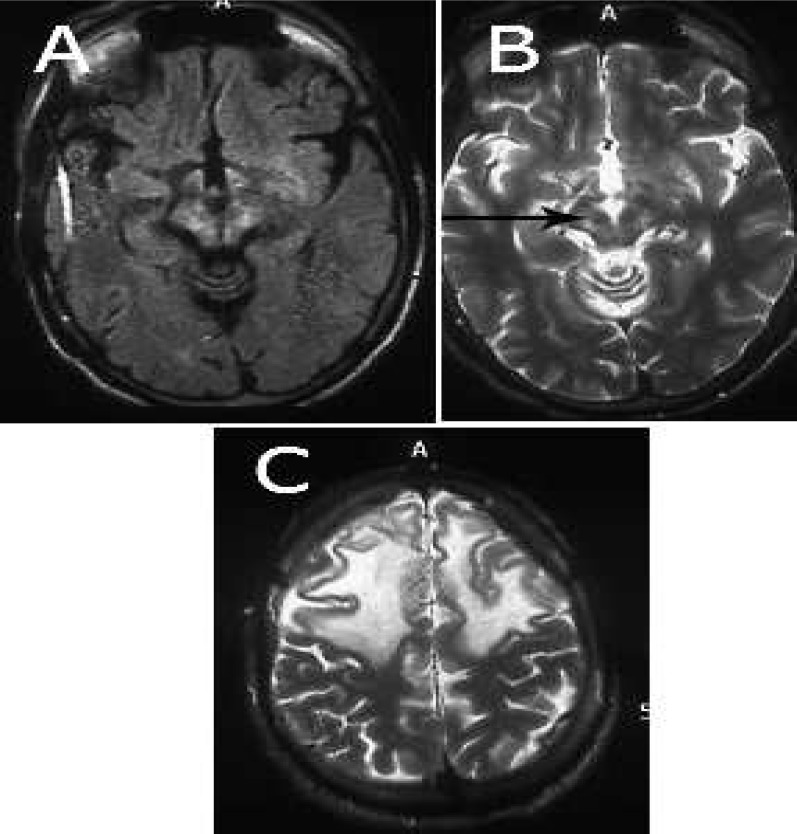
(a) Symmetrical hyperintensity on T2 weighted and fluid attenuated inversion recovery images over bilateral thalami, basal ganglia, claustrum, and dorsal mesencephalon, (b) giant Panda face, (c) T2 hyperintensity bilateral frontal white matter

**Table 1:** Diagnostic criteria for Wilson’s disease as advocated by Steinlieb^4^

Low serum ceruloplasmin levels (< 20 mg%)
Kayser–Fleischer rings in eyes
High liver copper levels (> 250 µg/g dry wt)
High 24 h urinary copper levels (> 100 µg/d or > 1.6 µmol/d)
Radioisotope copper studies using 64Cu, 67Cu or 65Cu, which assesses ability to incorporate copper into ceruloplasmin

The white matter abnormalities can occur in both pyramidal and extrapyramidal systems and can be symmetrical or asymmetrical. The white matter areas that are predominantly affected are dentatorubrothalamic, pontocerebellar, and corticospinal.^5^ These extensive white matter abnormalities are not common and are reported rarely in literature.

## Conclusion

A high index of suspicion is required for WD while dealing with young adults with extrapyramidal signs and neurobehavioral abnormalities with typical neuroradiological features. The radiological features may also show extensive gray and white matter abnormalities. Hence, patients with WD should also be evaluated for these abnormalities.

## Notes:

**How to cite this article: **Goyal S, Dabla S, Sharma B, Singh J, Yadav K. Wilson’s disease presenting with unusual radiological features. Iran J Neurol 2015; 14(3): 177-9.
